# Dendritic distribution of synaptic input creates a trade-off between input selectivity and flexibility

**DOI:** 10.1186/1471-2202-16-S1-P220

**Published:** 2015-12-18

**Authors:** Michiel Remme, Susanne Schreiber

**Affiliations:** 1 Institute for Theoretical Biology, Humboldt University, Berlin, 10115, Germany

## 

Information processing by cortical pyramidal neurons is shaped by the spatial distribution of synapses across the dendrites. A prominent hypothesis is that synapses with similar selectivities cluster on dendritic branches. This enables cooperative interactions between neighboring synapses through activation of voltage-dependent membrane currents, and helps establish independent integrative subunits, thereby expanding the computational power of a single neuron [1]. Some recent in vivo recordings argue against this hypothesis, suggesting that inputs that share the same stimulus selectivity are randomly distributed throughout the dendritic tree (e.g., [2]). Other studies seem to support clustered configurations of input selectivities, showing for example that the activity of a synaptic input is more strongly correlated with its neighbors than with more distant inputs [3].

One fundamental feature of the nervous system that has received little attention in this ongoing discussion is learning; specifically, how is the ability to change a neuron's selectivity (i.e., its flexibility) affected by the spatial distribution of synapses? This is highly relevant because the selectivity of many cortical pyramidal neurons is subject to ongoing modification, not only during development, but throughout adulthood (e.g., [4]).

Here, we show that the distribution of synapses across active dendrites shapes both the stimulus selectivity of the neuron, as well as the flexibility of the neuron's selectivity. Using cable theoretic analysis and numerical simulations of detailed neuron models we show that synapses that are randomly distributed across the dendrites allow for a modest stimulus selectivity that can be flexibly modified through synaptic plasticity. In contrast, a clustered distribution of synapses that encode the same stimulus allows for very strong stimulus selectivity, however, it hinders adjustment of this selectivity through synaptic plasticity, which requires slow and metabolically costly rearrangement of synaptic projections. Hence, the distribution of synapses across active dendrites creates a trade-off between selectivity and plasticity. We suggest that pyramidal neurons with different functions in the cortical information processing hierarchy exploit different ends of this spectrum.
